# Event-Related Potentials (ERP) Indices of Motivation during the Effort Expenditure for Reward Task

**DOI:** 10.3390/brainsci10050283

**Published:** 2020-05-08

**Authors:** Julie Giustiniani, Magali Nicolier, Juliana Teti Mayer, Thibault Chabin, Caroline Masse, Nathan Galmès, Lionel Pazart, Benoit Trojak, Djamila Bennabi, Pierre Vandel, Emmanuel Haffen, Damien Gabriel

**Affiliations:** 1 Department of Clinical Psychiatry, University Hospital of Besançon, 25000 Besançon, France; mnicolier@chu-besancon.fr (M.N.); jtetimayer@chu-besancon.fr (J.T.M.); cmasse@chu-besancon.fr (C.M.); djamila.bennabi@univ-fcomte.fr (D.B.); pierre.vandel@univ-fcomte.fr (P.V.); emmanuel.haffen@univ-fcomte.fr (E.H.); 2 EA 481, Laboratory of Neurosciences, University of Burgundy Franche-Comté, 25000 Besançon, France; tchabin@edu.univ-fcomte.fr (T.C.); nathan.galmes@edu.univ-fcomte.fr (N.G.); lpazart@chu-besancon.fr (L.P.); dgabriel@chu-besancon.fr (D.G.); 3 Clinical Investigation Centre, University Hospital of Besançon, Inserm CIC 1431, 25000 Besançon, France; 4 Neuroimaging and neurostimulation department Neuraxess, University of Burgundy Franche-Comté, 25000 Besançon, France; 5 FondaMental Foundation, 94000 Créteil, France; 6 Department of Psychiatry and Addictology, University Hospital of Dijon, 21079 Dijon, France; benoit.trojak@chu-dijon.fr; 7 EA 4452, LPPM, University of Burgundy Franche-Comté, 21000 Dijon, France

**Keywords:** motivation, effort, EEfRT, P300, SPN

## Abstract

Dynamic and temporal facets of the various constructs that comprise motivation remain to be explored. Here, we adapted the Effort Expenditure for Reward Task, a well-known laboratory task used to evaluate motivation, to study the event-related potentials associated with reward processing. The Stimulus Preceding Negativity (SPN) and the P300 were utilized as motivation indicators with high density electroencephalography. The SPN was found to be more negative for difficult choices compared to easy choices, suggesting a greater level of motivation, at a neurophysiological level. The insula, a structure previously associated with both effort discounting and prediction error, was concomitantly activated during the generation of the SPN. Processing a gain significantly altered the amplitude of the P300 compared to an absence of gain, particularly on centroparietal electrodes. One of the generators of the P300 was located on the vmPFC, a cerebral structure involved in the choice between two positive results and their predictions, during loss processing. Both the SPN and the P300 appear to be reliable neural markers of motivation. We postulate that the SPN represents the strength of the motivational level, while the P300 represents the impact of motivation on updating memories of the feedback.

## 1. Introduction

Motivation is a concept composed of various constructs such as goals, self-schemas, and interests [1]. Motivation, as a necessary element to the pursuit of a goal, is frequently reduced to goal-directed behaviors such as effort put forth into actions performed to obtain the expected results [2]. While motivation can trigger an activity, it is also used to perpetuate the same activity over periods of time [3]. In cognitive neuroscience, motivation is defined as neural representations of expected outcomes that predict decisions regarding effort investment [2]. In this way, motivation is strongly related to the anticipation of emotions and, more precisely, is influenced by the expected emotional consequences of one’s decision [4]. Based on these definitions, several methods of evaluating motivation have been developed via psychometric testing and laboratory tasks. While self-reports measure a relatively stable characteristic, they are subjective and cannot be directly connected to biological models of motivation. In comparison, laboratory tasks allow for an objective tool to correlate the behavior with the biological information collected. 

### 1.1. Previous Neuroimaging Studies on Motivation 

Several previous studies using different effort tasks, both cognitive and physical, showed the involvement of the insula and prefrontal cortex in motivational processes [5,6,7,8]. Insula activation has been demonstrated to be involved in processing response costs [9] and reward-dependent prediction errors [10]. More specifically, in an effort-based decision-making task, the insula codes the anticipated expense of energy [11]. The ventromedial prefrontal cortex (vmPFC) is involved in the decision-making process between two positive results and the associated predictions [12]. Reflecting the output of a choice comparison process [13] between different positive conditions to gain the greater reward [14], the primary value of the vmPFC process is in the guidance or motivation of the execution of a behavior [5]. The insula, on the other hand, is primarily involved in weighing the cost of the expected value by accounting for the effort to be made [5]. Dopaminergic manipulation coupled with positron emission tomography scan (PET scan) has previously implicated a mediating role in motivation and cost/benefit decision-making in the insula, striatum, and prefrontal cortex [15]. Further investigations on various clinical populations known for their motivational impairment corroborate the role of the striatal region in the motivational process [16,17], lending a common base to the same deficit displayed in different disorders. The recording of cerebral activity at rest, as measured by electroencephalography (EEG), showed that subjects who had a heightened propensity to effortful behavior had greater left frontal lobe activity [18]. In accordance with the approach-avoidance motivation systems [19] and their lateralization, the left prefrontal cortex is associated with the pursuit of approach-related goals [20], suggesting that participants with increased left frontal lobe activity would be more willing to expend a greater effort in the pursuit of larger rewards [18]. While the neural structures involved in motivational processes occurring during effort expenditure for a reward have been investigated, the temporal sequence of these responses to motivation has yet to be elucidated. Indeed, motivation is a dynamic construct with various successive, interrelated processes occurring close together. Neither functional magnetic resonance imaging (fMRI) [5,6,7,8], PET scan [15], nor resting state activity at the EEG [18] allows for the distinction to occur with sufficient temporal resolution cerebral structures to determine each cognitive step. Since motivation is strongly associated with the reward experience, the temporal course of motivation can be measured by focusing on reward processing. The complex reward construct is characterized by distinct processes, namely outcome processing, reward learning, and reward anticipation [21,22]. Using neuroimaging methods with high temporal resolution may, therefore, be helpful in distinguishing the role of motivation in each process. The advantage provided by the millisecond temporal resolution of event-related potentials (ERP) may be particularly relevant in exploring the dynamic aspects of the motivational process while limiting confusion with other processes. Whereas behavioral performances bring motivational information in terms of global costs that subjects are willing to overcome, an ERP analysis allows the ability to examine other aspect of motivation, such as the anticipation of emotions and impact on outcome processing [4].

Previous studies have suggested that several ERPs may be closely related to motivation. The first one is the stimulus preceding negativity (SPN), a non-motor expectancy wave that precedes a relevant stimulus during which a non-motor response was required [23]. More precisely, SPN has been described as a slow cortical potential that becomes negative in anticipation of relevant events and reflects anticipatory attention [24]. The SNP has also been interpreted as an anticipation of the affective motivational valence of stimuli, with the largest amplitude occurring in anticipation of one relevant stimulus [24,25]. Given the temporal course of the SPN, this potential appears to be associated to the reward anticipation, or more precisely the “wanting” [21], reflecting the neural representation of the reward anticipation, with the insula identified as the main generator [26,27,28,29] of this potential. The second ERP is the P300, a positive ERP peaking between 300-600 ms post-stimulus with largest amplitude at centro-parietal scalp sites [30]. P300 is frequently associated with motivation [31], with an amplitude described as proportional to the motivational level [32,33,34,35,36]. The P300 occurs following the reward and is associated to the reward outcome and its hedonic impact [21]. 

### 1.2. Evaluation Methods

Previous neuroimaging studies on motivation have used various laboratory tasks. In laboratory tasks, the construct of motivation must be converted in terms of a measurable, assessable behavior, such as the effort to obtain rewards. Various designs have been created to evaluate motivation as effort-based decisions, with many falling into the categories of “HandGrip” tasks [37,38], “Button Press” tasks [39,40,41], or cognitive tasks [42]. The Effort-Expenditure for Rewards Task (EEfRT) is the most frequently used of the Button Press tasks and was originally created to study motivation deficits in individuals diagnosed with neuropsychiatric disorders [39]. The EEfRT is a multi-trial task in which participants are asked to choose between two options, one easy and one difficult, as a function of the magnitude of the monetary reward and the probability of receiving the reward if the task is successfully completed [39]. It is important to note that the difficulty of the task is related to a physical effort and therefore does not involve any cognitive processes beyond the motivation to complete the task. More specifically, in the EEfRT, subjects must evaluate various costs such as effort, uncertainty, and delays, with the intention of promoting behaviors that may yield larger rewards. The choice of manipulating probability during the EEfRT is motivated by the notion that probability is sensitive, like other costs, to dopaminergic functioning [43,44]. Consequently, the proportion of difficult choices, reported to the probability condition, is designed to reflect motivational level. In EEfRT, subjects who choose the difficult option at low probability conditions are considered to be highly motivated as they are willing to overcome a greater cost. The EEfRT was primarily developed to evaluate motivational dysregulation in clinical populations [45] and its use has been validated on several different populations such as those with mood disorders [46], schizophrenia [47], obesity [48], and in cannabis users [49], demonstrating its acceptability. 

### 1.3. Study Rationale

The current study adapted the EEfRT to ERP recordings to explore the dynamic and temporal aspects of motivation in healthy volunteers. Our preliminary step was to check that this adaptation remains a para-clinical evaluation tool and that changes made for ERP recordings would not alter the task or the performance of the EEfRT. Following that step, the primary objective was to validate the presence of the P300 and SPN during outcome processing and to identify the specificities of each component. We also sought to investigate the relationship of their amplitudes to the motivation measured during the EEfRT. For comparison, we also analysed the Feedback Related Negativity (FRN), which is associated with the early outcome processing. This ERP affirmed the early processing of outcomes on the basis of a binary classification of good or bad outcomes [36,50,51]. Using high-resolution electroencephalography technology, we also sought to identify the brain structures involved in ERP generations, especially the insula which has been suspected in the generation of the SPN. This study will help better understand individuals’ motivation impairment in a natural setting, before continuing the investigation in pathological populations. The adaptation of ERP recording would allow a more complete, neurophysiological understanding of the role of motivation in effort-based decision making while focusing on the reward anticipation with the SPN and reward processing with P300.

## 2. Methods

### 2.1. Participants

Twenty healthy male volunteers were recruited to participate in the current study (mean age 23.7 ± 3.2 years). All were right-handed (assessed with the Handedness Questionnaire of Oldfield, 1971) and over 18 years old. No participants had any previous medical history of psychiatric disorders, substance abuse, alcohol abuse, neurological diseases, traumatic brain injury, or stroke nor were any participants taking any medication at the time of the study. The presence and intensity of anhedonia were evaluated on all participants with the Chapman Anhedonia Scale, which assess physical and social anhedonia (average: 14 ± 4.3 and 7.2 ± 4.4, respectively) and the Snaith-Hamilton Pleasure Scale (SHAPS) was used to assess the hedonic response (average: 13 ± 0.9). These scores preclude the presence of any form of anhedonia in our participants. In addition, participants completed the Big Five Inventory-French (BFI-Fr) to control for the impact of personality traits on performance [52]. All participants were compensated for their participation. Furthermore, every patient provided written informed consent prior to enrolment. The study procedures were clearly explained to the participants and they were given the opportunity to ask questions. All methods were performed in accordance with the relevant guidelines and regulations and all methods were approved by the Ethics Committee of Besançon University Hospital (authorization given by the General Health Administration (ANSM 2016-A00870-51)).

### 2.2. Experimental Task and Measurements 

The experiment began with a calibration phase, consisting of determining the maximum number of button presses participants were able to perform in 7 s with the index finger of their right hand and in 14 s with the auricular finger of the left hand, allowing for personalization of the difficulty of the EEfRT [53]. 

The EEfRT was modified based on the original version [39] and adapted for ERP analysis. The goal of the EEfRT is to win as much money as possible by completing either easy or hard tasks. Each task is selected as a function of the amount of money that can be won if the task is completed and the probability of receiving the reward when the task is completed.

In the adaptation used in the current study, the number of trials was fixed to 120. To complete the easy task, participants had to execute 70% of their maximum number of buttons presses obtained with the right index finger in the calibration phase within a limited time of 7 s. When the easy task was completed, participants were eligible to win 1 EUR. For the hard task, participants were required to execute 90% of their maximum number of buttons presses obtained in the calibration phase with the auricular finger of the left hand within the limited time of 14 s. The time assigned for the completion of the hard task was reduced compared to the original task (21 s) in order to compensate for the increased number of trials, which increase the study time. When the hard task was completed, participants were eligible to win an amount of money in a range of 1.5, 3, 4.5, or 6 EUR. Probabilities to win the money when the task was completed were of 10%, 50%, or 90%. These probabilities applied to both the hard task and the easy task and were distributed in equal proportions across the experiment. 

**Figure 1:**
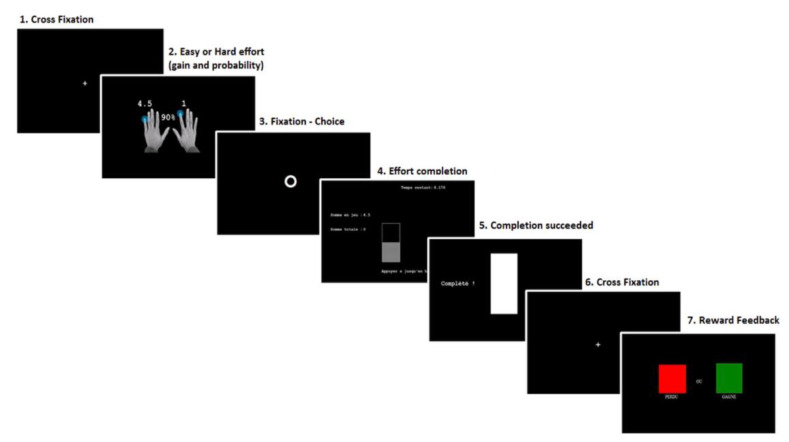
Schematic diagram of a trial in the altered version of the EEfRT. 1. Begin with a cross fixation of 1 s; 2. Presentation of the probability to receive the reward associated with the amount of the reward for easy and hard effort conditions (1 s); 3. Circle of maximum 10 s for the selection of easy or hard effort; 4. Completion of the effort by button press; 5. Success screen; 6. Cross fixation of 1 s; 7. Reward feedback, the “rewarded” condition = green square and “not rewarded” condition = red square.

Figure 1 outlines the detailed trial sequence of our adaptation of the EEfRT. During the task, trials occurred according to the following steps: for 1 s, a first screen indicated the probability to receive the money after completion (10%, 50%, or 90%) and the amount of money at stake for an easy effort (invariably 1 EUR) and a hard (1.5, 3, 4.5, or 6 EUR) effort. A circle then appeared on the screen and participants were required to choose between the easy and the hard task. After the selection, participants had to quickly press buttons to fill a bar before their time ended. If they succeeded, a cross appeared on the screen for 1 s, followed by a feedback screen of 2 s indicating if the money was won (green square) or lost (red square) for that particular trial (reward screen). If they failed, a red square appeared on the screen for 2 s.

This adaptation of the EEfRT was programmed in E-prime (Psychology Software Tools Inc.; Sharpsburg, PA, USA). The probability and amount of order was randomized across participants. To ensure task comprehension, subjects received oral instructions and were provided with a series of task instruction, followed by a few practice trials prior to starting the experiment.

### 2.3. Data Acquisition 

EEG signals were recorded using a 256 channel Geodesic Sensor Net (Electrical Geodesics Inc.; EGI, Eugene, OR, USA). All channels were referenced to the vertex (Cz) and collected with a high impedance amplifier, Net Amp 300 amplifier (Electrical Geodesics) using Net Station 4.5 software (Electrical Geodesics). Data were recorded continuously with a high-pass filter at 1 Hz and a sampling rate at 1000 Hz. Subjects were instructed to limit body movements, eye blinks, and muscular contractions during task selection and reward feedback. 

### 2.4. Data Analysis 

EEG data analysis was performed using Cartool Software 3.55 [54]. Raw EEG data were re-referenced offline to a common average reference. Analyses were conducted on the interval around the reward screen for two intervals. The first temporal interval, computed for easy and hard tasks, was related to the SPN. Epochs of 700 ms (500 ms prior to the outcome to 200 ms after) were extracted from the raw data, with a baseline correction of 100 ms applied prior to the selection of an easy or hard task by the participant. The SPN was defined as the mean voltage within 200 ms prior to the reward feedback [55]. In the second temporal interval, epochs of 700 ms (100 ms prior to reward feedback to 600 ms following reward feedback) were extracted from the raw data and analyzed, with a baseline correction between 100 ms applied before the feedback to the onset of the feedback (100 ms to 0 ms). The P300 was defined as the mean voltage between 290 to 410 ms, based on grand averages of ERPs for “rewarded” and “not rewarded” conditions. An additional analysis of the FRN, defined as the mean voltage from 240 to 290 ms, was conducted.

For all ERPs, a band pass filter was applied between 1 to 30 Hz and a notch filter was applied at 50 Hz to remove environmental artifacts. A semi-automatic artifact rejection method was used, with a fixed criterion of ±100 µV. Remaining epochs were visually inspected, manually removing those containing blinks, eye movements, or other sources of transient noise from the analysis, using a restrictive approach. The average percentage of easy and hard trials rejected for the analysis of the SPN was, respectively, 43% and 47%. The average percentage of win and loss trials rejected for the analysis of the P300 was, respectively, 36% and 39%. Electrodes with an aberrant signal (excessive noise due to malfunctioning or a bad signal during data collection) were interpolated using a three-dimensional spline algorithm (average: 4.67% interpolated electrodes [56]). Based on the previous literature on feedback processing, six central electrodes (Fpz, Fz, FCz, Cz, CPz, Pz) were chosen for analysis [50,51,57,58,59,60,61].

### 2.5. Source Imaging

Source localization was applied using a distributed linear inverse solution based on a Local Auto-Regressive Average (LAURA) model, comprising a solution space of 3005 nodes to estimate the brain regions in response to the different electrocortical map configurations. The current distribution was calculated within the grey matter of the average brain provided by the Montreal Neurological Institute (MNI).

Source reconstruction was performed for the SPN and the P300 to accurately determine the neural sources of motivational states. The entire brain response was analyzed and brain regions showing differences in activity between the anticipation of an easy and a hard task (for the SPN), as well as between a reward and an absence of reward (for the P300), were compared during their respective time windows.

## 3. Results

### 3.1. Behavioral Results on the EEfRT

Participants’ press rate was, on average, 83 (±11) on the difficult task and 40 (±5) on the easy task. There was an average of 11.3 (±11.0) and 2.6 (±2.6) non-completed trials on the difficult and the easy tasks, respectively. Individual measures of difficulty were adapted to each participant, because the number of button presses on the difficult task was not found to be associated with to the number of completed trials (*r_s_* = 0.31, *p* = 0.17), nor to the number of times participants had selected the difficult task (*r_s_* = −0.02, *p* = 0.94) at the end of the EEfRT.

Table 1 shows the average number (±standard deviation) of wins and losses for the different probabilities of the easy and hard tasks. Consistent with previous studies using the original version of the EEfRT, reward magnitude strongly impacted the selection of task’s difficulty, as indicated by a one-way repeated measures ANOVA with amount (1.5, 3, 4.5, or 6 EUR) as a within subject factor and the number of hard choices as the dependent variable (*F*(3,57) = 165.726, *p* < 0.0001). The higher the reward, the more likely participants were to try to win the reward by selecting hard choices. This effect was statistically significant across all conditions (*p* < 0.0001 for all, Bonferroni corrected), except between 4.5 and 6 EUR (*p* = 0.25).

**Figure 2:**
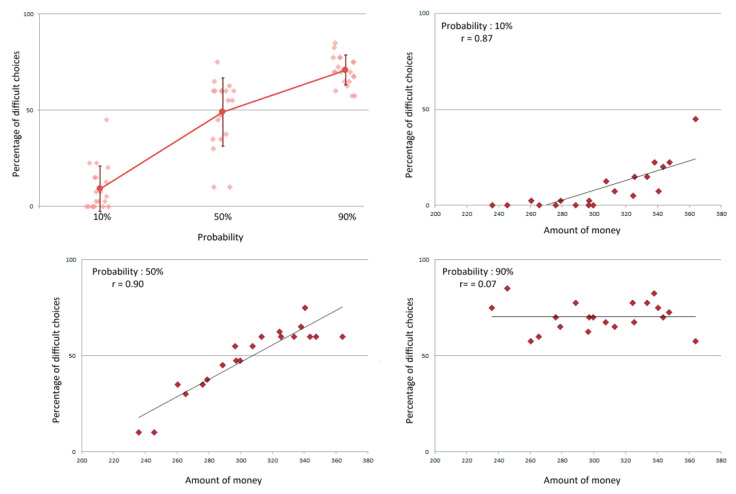
Top left: Percentage of difficult choices according to the probability of gain. For each probability, individual performances are represented in addition to the mean percentage and its standard deviation. Other distributions represent the individual percentage of difficult choices as a function of the money participants attempted to win at the 10% (top right), 50% (bottom left) and 90% (bottom right) conditions. Spearman r values are notated for each condition.

A significant relationship was also observed between the probability to win the money upon completion of the task and the selection of task difficulty, as shown by a one-way repeated measures ANOVA with probability (10%, 50%, or 90%) as a within subject factor and the percentage of hard choices as the dependent variable (*F*(2,38) = 140.329, *p* < 0.0001) (see Figure 2). Significant differences in the proportion of hard choices were present across all probability conditions (all Bonferroni corrected *ps* < 0.0001). For a high probability of gain, the proportion of hard choices was significantly higher than for an equal or a low probability of gain. While the proportion of hard choices was significantly higher than the proportion of easy choices for a high probability of gain and lower for a low probability of gain (both Bonferroni corrected *ps* < 0.0001), there was no difference when there was an equal probability of gain (50%). It is at this probability that the largest variability was observed among participants, with the proportion of hard choices ranging between 10% and 75% (see Figure 2). 

A strong marker of motivational state was indicated by measuring the proportion of hard choices the participants selected. Namely, a strong relationship was found between the total amount of money participants attempted to win and the proportion of hard choices at 10% (*t*(18) = 7.64, *p* < 0.0001) and 50% (*t*(18) = 8.99, *p* < 0.0001, see Figure 2). No such relationship was found when evaluating the proportion of hard choices at a probability of 90% (*t*(18) = 0.31, *p* = 0.76, see Figure 2). 

Self-rating scales failed to demonstrate a difference in hedonic responses and in the presence of anhedonic symptoms. There was no correlation found between behavioral performances and scores on either the Chapman Anhedonia Scale [62] or the Snaith-Hamilton Pleasure Scale (SHAPS) [63]. In addition, there was no correlation between behavioral performances and any of the personality traits evaluated with the Big Five Inventory-French (BFI-Fr) [52].

### 3.2. ERP Results on the EEfRT

#### 3.2.1. Reward Anticipation

**Figure 3:**
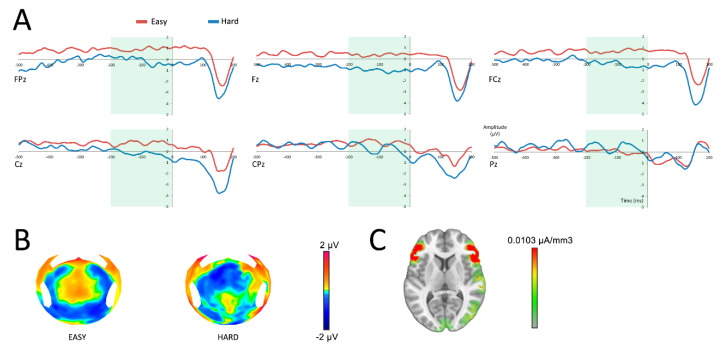
Neural responses before the reward feedback (at 0 ms). **A**: reward anticipation after having successfully completed an easy (red) or a hard (blue) task in six central surface electrodes. The time period of analysis of the SPN is presented in light green. **B**: topographic representation of the anticipation of an easy or a hard task in the time frame of the SPN. **C**: source localization of the difference between both conditions in the time frame of the SPN.

To determine whether the anticipation of the reward was different following the selection of an easy or a hard task, SPN data were analyzed using a repeated measures ANOVA, with repeated measures variables being difficulty (easy/hard) and electrodes (FPz, Fz, FCz, Cz, CPz, Pz). Analysis of the SPN demonstrated a main effect of difficulty on the amplitude of the SPN (*F*(1,19) = 5.38; *p* = 0.03), with a more negative response for hard choices (Figure 3). No interaction was found between the variables difficulty and electrodes (*F*(5,95) = 1.50; *p* = 0.24).

Source reconstruction revealed a main activation in the vicinity of the anterior insula and the lateral prefrontal cortex, as well as an activation of the right temporo-parietal area (Figure 3C). Because the insula is well known as the main generator of the SPN [26,27,28,29], we made the hypothesis that the main activation observed was originating from this area, with an approximation due to the low spatial resolution of LAURA. To focus on the insula, Cartool software offers the opportunity to select a list of Talairach regions and generates a group of solution points (nodes) that fit within each of the named regions. Activations of the insula were compared following an easy and a hard task within the time window of the SPN. To allow current density measures (indicating activation strength in µA/mm^3^) to be extracted from the insula, the inverse solution was estimated for the group of solution points for each time window of interest. A source analysis performed on the same time window as the SPN revealed a main effect of difficulty on the amplitude of the insula (*t*(19) = −4.19; *p* < 0.001). 

#### 3.2.2. Outcome Processing

A repeated measures ANOVA was used to analyze the processing of the outcome for both the FRN and the P300, with repeated measures variables being outcome (win/no win) and electrodes (FPz, Fz, FCz, Cz, CPz, Pz).

**Figure 4:**
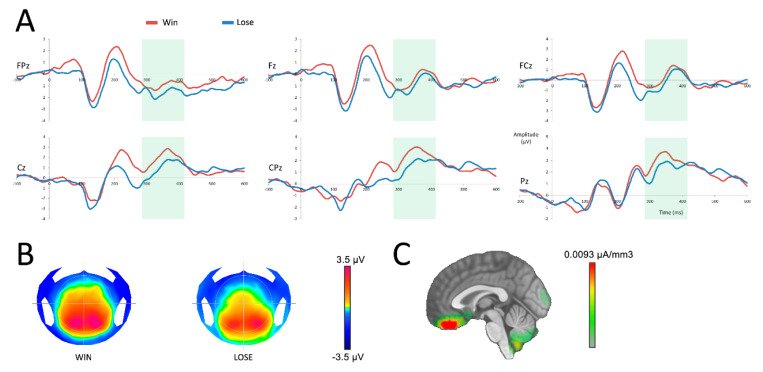
Neural responses after the reward feedback (at 0 ms). (**A**) reward processing after a win (red) or a loss (blue) in six central surface electrodes. The time period of analysis of the P300 is presented in light green. (**B**) topographic representation of a win and a loss in the time frame of the P300. (**C**) source localization of the difference between both conditions in the time frame of the P300.

The P300 analysis demonstrated a main effect of the outcome (*F*(1,19) = 7.47; *p* = 0.01), with a more negative response when participants received no reward. No interaction was found between the variables outcome and electrodes (*F*(5,95) = 1.05; *p* = 0.36). Source localization performed on the P300 revealed that more negative signals observed in the absence of a reward emerged primarily from the vmPFC (see Figure 4 C). Source analysis comparing the activity of the vmPFC (specifically the bilateral rectal and mid orbital gyri) after a reward and an absence of reward in the time window of the P300 did not yield a significant difference (*t*(19) = 1.04; *p* = 0.31). 

The analysis of the FRN demonstrated a main effect of the outcome (*F*(1,19) = 44.3; *p* < 0.00001), with neural responses being more negative after a loss than after a win. There was also a trend showing a more negative FRN for the most central electrodes (*F*(5,95) = 3.19; *p* = 0.06 after Greenhouse–Geisser adjustment).

We also tested the difficulty’s effect on the processing of the outcome with a repeated measures ANOVA on the time frame of the P300, with repeated measures variables being difficulty (easy/hard) and electrodes (FPz, Fz, FCz, Cz, CPz, Pz). Neither a main effect of the difficulty (*F*(1,19) = 1.22; *p* = 0.27) nor on interaction between difficulty and electrodes (*F*(5,95) = 1.43; *p* = 0.21) were found.

### 3.3. Relationship between Behavioral and Electrophysiological Results

With an exploratory approach, we aimed to assess the relationship between behavioral and electrophysiological motivational data. We compared amplitudes during outcome anticipation (SPN and insula) and outcome processing (P300) with the behavioral indicators of participants’ effort in decision making, the amount of money they finally won, and the percentage of difficult choices. 

The total amount of money was positively correlated to both the activity of the insula (the difference between an easy and a hard task) during outcome anticipation (*t*(18) = 2.70, *p* = 0.01) and the activity over the electrode Pz (the difference between positive minus negative outcomes) during information processing (*t*(18) = 2.16, *p* < 0.05). In addition, we examined whether there was a significant relationship between the total amount of money and the activity of the FRN (difference between a reward and no reward); however, there were no significant correlations (all *ps* > 0.05). 

Since we observed that, at a behavioral level, the proportion of hard choices selected by participants at both 10% and 50% may be a reliable indicator of participants’ motivational state, we postulated that it may be related to the activity of the insula and/or the P300. We also observed a strong relationship between the percentage of difficult choices (easy/hard) at a probability of 10% and the amplitude of P300 after both a positive (*t*(18) = 3.29, *p* = 0.004) and a negative (*t*(18) = 2.84, *p* = 0.01) outcome on Cz.

## 4. Discussion

The primary goal of the current study was to explore the neural dynamic of motivation using an adaption of the EEfRT on the analysis of ERPs. Based on previous studies suggesting that both the SPN and P300 may be reliable markers of motivation, the present study aimed to confirm this notion by evaluating their time course as well as the direct relationship between these ERPs and the physical effort made to obtain a reward. 

The first step was to validate that the adapted EEfRT produced similar results as the original version. As expected [39], we observed that the probability to receive the reward strongly impacted the decision to select easy or hard choices; the higher the probability to get the reward, the higher the proportion of hard choices and, conversely, the lower the probability to get the reward, the higher the proportion of easy choices. Similarly, when there was a middle probability to receive the gain, there was no significant difference in the selection of choice difficulty, with subjects demonstrating a greater heterogeneity in their choices under this condition. The proportion of hard choices made when there was a low or middle probability of gain was strongly related to the total amount of money at the end of the experiment, but not when there was a high probability of gain. These results are in accordance with previous studies of pharmacologically induced motivation by d-amphetamine [64]. Indeed, an administration of d-amphetamine has been shown to enhance the number of difficult choices in both the middle and low probability conditions in healthy subjects [64]. Here, we did not observe any correlation between anhedonia scores and the proportion of hard choices, likely due to our healthy populations and the overall low scores for this psychometric test. These differences suggest that the middle probability condition may be more sensitive to detect a pathological lack of motivation, whereas the low probability condition is more likely to better discriminate between subjects as a function of the strength of their motivation. These behavioral results confirmed that the few changes in the EEfRT, adapted for its ERP analysis, did not alter expected behaviors and measures in an equal manner to the effort-based decision making.

That participants were required to perform at a higher effort to obtain a greater reward at the EEfRT imparts a high motivational valence. A major issue to consider in the exploration of the neural representations of the motivation to get a reward is that the outcome process is composed of several dimensions, including reward anticipation, outcome processing, and reward learning [21,22]. The current study looks more deeply at the SPN as a function of reward anticipation. In accordance with previous studies showing that the possibility to receive desirable outcomes induces a greater anticipatory negativity [25], the current study demonstrated that the SPN was more negative for difficult choices compared to easy choices. Since there was a higher potential reward, it is likely that participants were more hopeful to obtain the desirable outcome. Therefore, this larger negativity during the outcome anticipation following a difficult choice neurophysiologically induces a greater level of motivation. The previous literature has demonstrated that these variations of amplitude not only reflect a modulation of attentional processes, but are primarily controlled by emotions [65,66]. 

The current study confirmed that the insula was concomitantly activated during the generation of the SPN. Previous studies have shown that the SPN provided temporal information on neocortical processes underlying the expectation [28] and have identified the insula as the main generator [26,27,28,29]. Furthermore, activity of the anterior insula has previously been associated with both effort discounting and prediction error signals [67]. The right anterior insula, in particular, appears to be a critical structure for awareness of visceral sensations [26], which may explain why insula activity was increased in subjects earning a larger amount of money at the end of the experiment. Therefore, it is plausible that the previously identified insula does, indeed, play a role in coding the anticipated expense of energy [11]. The insula is not only involved in the development and updating of motivational states, but also in cognitive control tasks as well as some tasks that elicit affective processing [68]. Consequentially, the insula has been frequently identified to make the link between affect and cognition. By utilizing psychometric controls, we were able to effectively the impact of personality traits on our results, as previous literature has identified personality traits as a potential bias [69,70].

The P300 was the second ERP closely related to motivation that was analyzed. This potential occurs following the receipt of the outcome and, in this way, can reflect outcome processing and reward learning. In that respect, the P300 appears as a complement to the SPN. Consistent with previous research [71,72,73], processing a gain significantly altered the amplitude of the P300 compared to the absence of a gain, particularly on centroparietal electrodes [35,72]. Since the amount of attention paid to the stimulus is believed to influence the P300 response [74], it has been suggested that motivation may increase the significance of relevant stimuli. More precisely, the P300 has been attributed to working memory processing following unexpected events [74,75] and may reflect the adjustment of behavior [76]. Indeed, the P300 can provide a measure of participant engagement in the task. Moreover, a significant positive correlation was found between P300 amplitude and the proportion of difficult choices at the low probability condition, which we behaviorally described as the condition better suited to reflect the strength of motivation. The correlation between P300 amplitude and proportion of difficult choices at this low probability suggests that the P300 does not reflect the outcome processing only. Indeed, if the P300 amplitude was only correlated to the outcome processing, this should have been observed at the high probability condition. Motivation has been defined as a cognitive mechanism which generates a modification of other cognitive functions, such as attention and working memory [77]. As other studies have shown, it stands, then, that this component indicates a motivational level and is not just an indicator of outcome processing [31,35,36,78]. Furthermore, P300 amplitude has been shown to be inversely correlated to motivation in a clinical population, such as apathy [79] or anhedonia [80]. Our results being exploratory, no adjustment for multiple correlation having been made, a replication on a larger sample of participants will be necessary to confirm such a relationship.

Another novel finding of this study is the confirmation that one of the generators of the P300 wave is located in the frontal region [73,81,82], more precisely in the vmPFC during loss processing. The vmPFC is a cerebral structure involved in the choice between two positive results and in their predictions [12,83], more precisely in the decision between two appealing results [84]. In addition, attentional and motivational information is integrated into the vmPFC and are reciprocally modulated [85]. This activation reflects the evaluation of decision outcomes in order to adjust future behaviors accordingly [20].

In addition, we analyzed whether there was an FRN associated with the early processing of the result on the basis of a binary classification of good or bad outcomes [36,50,51]. We did not find any correlation between FRN amplitude and performances on the EEfRT. Without any motivational information, we confirmed the presence of an FRN that was associated to the early processing of the outcomes, with a larger amplitude after a loss than after a win [36,50,51].

As the current study evaluated motivation by focusing on the assumption that subject agrees to the overcome, several limitations need to be considered to evaluate these results. One limitation is that we averaged all easy and difficult choices, without consideration of the probability conditions, which are important given that the SPN is modulated by the magnitude of the outcome and the probability to receive the reward [25,86]. Since participants were more likely to select easy choices at the low probability condition and more difficult choices at the high probability condition, the probability factor may have influenced the SPN. This bias is difficult to bypass in our design without substantially increasing the number of trials, which would be counterproductive, due to likelihood of fatigue or the imposition of choices to subjects. Indeed, the EEfRT adapted to the ERP was developed to eventually become a para-clinical evaluation tool. Although we acknowledge this bias, it does not change the motivational aspect of the SPN recorded in our participants. One other limitation needs to be addressed. As previously noted, in the EEfRT the costs to overcome include delay, probability and effort. Indeed, the same substance drives to overcome an increased global cost such as effort [87], probability to receive the reward [43], and temporal delay to receive it [88]. It is, however, necessary to precisely note that each type of cost involves specific cerebrals structures [11,44,89,90]. In our paradigm, it is not the physical effort which is important, rather the global cost that subjects consented to overcoming. Unfortunately, we could not control the effect of the probability on FRN amplitude due to the limited number of trials in each condition, which limited the results. Some authors have previously described that the FRN may reflect prediction error and, in this way, could be impacted by probability [76]. More precisely, the FRN was affected by the risk, with a reduced amplitude for positive feedback conditions [91]. The lack of probability control on the FRN should therefore be considered as a limitation to our results. In addition, the effort expended may be influenced by the FRN and, more specifically, its avoidance could be influenced by reward processing [92]. However, our design is such that once the choice made, the effort associated with choosing cannot be avoided. The absence of influence of the task difficulty on its amplitude allowed us to conclude that only the SPN and P300 were specific to the motivation. One other potential limitation is that all participants in this study were young adult men, limiting the ability to expand these results to the whole population. However, a homogenic population provided for the ability to avoid confusion caused by gender, such as the menstrual cycle in female participants, which may influence emotional processing [93]. Finally, more technical aspects of the study may be considered as limitations. First, filtering parameters used in the present study are debated, some authors arguing that any high-pass filters > 0.3 Hz should be avoided in ERP research [94]. We wanted to keep the same filters as in our previous studies [73], for comparison purpose. Second, the accuracy of source reconstruction could have been improved by digitizing the electrodes for each participant, or by reconstructing sources with participant’s anatomical MRI. Third, figures from the present study suggest that FRN and P300 responses have different time latency in the frontal and parietal cortical areas. A reanalysis of ERP responses separately for frontal and parietal electrodes may then reveal how ERPs responses propagate when the outcome is processed.

In conclusion, we confirmed that both the SPN and the P300 are reliable neural markers of motivation. We were able to show that they are complementary and induce different aspects of motivation. While the SPN represents the strength of the motivational level or the willingness to make an effort, the P300 is associated with late feedback processing, reflecting the impact of motivation on updating memories of the feedback. Each ERP constitutes a potential target for the identification of endophenotypes in neuropsychiatric disorders. Indeed, EEfRT was primarily developed to evaluate the dysregulation of motivational processes, a key contributor to various psychopathologies [45]. In the future, coupling the ERP analysis to the EEfRT may allow a better understanding in various clinical populations given the same motivational impairment is associated with a similar or dissimilar cerebral activity. 

## Figures and Tables

**Table 1:** Average number (±standard deviation) of wins and losses for the different probabilities of receiving the reward in the easy and hard tasks.

	Easy Task			Hard Task		
	**10%**	**50%**	**90%**	**10%**	**50%**	**90%**
**W** **in**	3.8 ± 0.5	10.6 ± 3.4	10.7 ± 3.0	0.2 ± 0.5	9.5 ± 3.4	25.4 ± 3.0
**Loss**	32.6 ± 4.3	10.1 ± 3.8	1.3 ± 0.6	3.4 ± 4.3	10.0 ± 3.8	2.8 ± 0.6

## References

[1] Murphy P.K., Alexander P.A. (2000). A Motivated Exploration of Motivation Terminology. Contemp. Educ. Psychol..

[2] Braver T.S., Krug M.K., Chiew K.S., Kool W., Westbrook J.A., Clement N.J., Adcock R.A., Barch D.M., Botvinick M.M., Carver C.S. (2014). Mechanisms of motivation-cognition interaction: Challenges and opportunities. Cogn. Affect. Behav. Neurosci..

[3] Schunk D.H. (2000). Coming to Terms with Motivation Constructs. Contemp. Educ. Psychol..

[4] Mellers B.A., McGraw A.P. (2001). Anticipated Emotions as Guides to Choice. Curr. Dir. Psychol. Sci..

[5] Chong T.T.-J., Apps M., Giehl K., Sillence A., Grima L.L., Husain M. (2017). Neurocomputational mechanisms underlying subjective valuation of effort costs. PLoS Biol..

[6] Schmidt L., Lebreton M., Cléry-Melin M.-L., Daunizeau J., Pessiglione M. (2012). Neural mechanisms underlying motivation of mental versus physical effort. PLoS Biol..

[7] Croxson P.L., Walton M.E., O’Reilly J.X., Behrens T.E.J., Rushworth M.F.S. (2009). Effort-based cost-benefit valuation and the human brain. J. Neurosci. Off. J. Soc. Neurosci..

[8] Engström M., Karlsson T., Landtblom A.-M., Craig A.D.B. (2014). Evidence of Conjoint Activation of the Anterior Insular and Cingulate Cortices during Effortful Tasks. Front. Hum. Neurosci..

[9] Kuhnen C.M., Knutson B. (2005). The Neural Basis of Financial Risk Taking.

[10] Pessiglione M., Seymour B., Flandin G., Dolan R.J., Frith C.D. (2006). Dopamine-dependent prediction errors underpin reward-seeking behaviour in humans. Nature.

[11] Prévost C., Pessiglione M., Météreau E., Cléry-Melin M.-L., Dreher J.-C. (2010). Separate valuation subsystems for delay and effort decision costs. J. Neurosci. Off. J. Soc. Neurosci..

[12] Jensen J., Smith A.J., Willeit M., Crawley A.P., Mikulis D.J., Vitcu I., Kapur S. (2007). Separate brain regions code for salience vs. valence during reward prediction in humans. Hum. Brain Mapp..

[13] Shenhav A., Straccia M.A., Botvinick M.M., Cohen J.D. (2016). Dorsal anterior cingulate and ventromedial prefrontal cortex have inverse roles in both foraging and economic choice. Cogn. Affect. Behav. Neurosci..

[14] Blair K., Marsh A.A., Morton J., Vythilingam M., Jones M., Mondillo K., Pine D.C., Drevets W.C., Blair J.R. (2006). Choosing the lesser of two evils, the better of two goods: Specifying the roles of ventromedial prefrontal cortex and dorsal anterior cingulate in object choice. J. Neurosci. Off. J. Soc. Neurosci..

[15] Treadway M.T., Buckholtz J.W., Cowan R.L., Woodward N.D., Li R., Ansari M.S., Baldwin R.M., Schwartzman A.N., Kessler R.M., Zald D.H. (2012). Dopaminergic mechanisms of individual differences in human effort-based decision-making. J. Neurosci. Off. J. Soc. Neurosci..

[16] Yang X., Huang J., Lan Y., Zhu C., Liu X., Wang Y., Cheung E.F.C., Xie G., Chan R.C.K. (2016). Diminished caudate and superior temporal gyrus responses to effort-based decision making in patients with first-episode major depressive disorder. Prog. Neuropsychopharmacol. Biol. Psychiatry.

[17] Huang J., Yang X.-H., Lan Y., Zhu C.-Y., Liu X.-Q., Wang Y.-F., Cheung E.F.C., Xie G.-R., Chan R.C.K. (2016). Neural substrates of the impaired effort expenditure decision making in schizophrenia. Neuropsychology.

[18] Hughes D.M., Yates M.J., Morton E.E., Smillie L.D. (2015). Asymmetric frontal cortical activity predicts effort expenditure for reward. Soc. Cogn. Affect. Neurosci..

[19] Spielberg J.M., Miller G.A., Warren S.L., Engels A.S., Crocker L.D., Banich M.T., Sutton B.P., Heller W. (2012). A brain network instantiating approach and avoidance motivation. Psychophysiology.

[20] Spielberg J.M., Stewart J.L., Levin R.L., Miller G.A., Heller W. (2008). Prefrontal Cortex, Emotion, and Approach/Withdrawal Motivation. Soc. Personal. Psychol. Compass.

[21] Berridge K.C., Robinson T.E., Aldridge J.W. (2009). Dissecting components of reward: ‘liking’, ‘wanting’, and learning. Curr. Opin. Pharmacol..

[22] Berridge K.C., Kringelbach M.L. (2015). Pleasure Systems in the Brain. Neuron.

[23] Brunia C.H., Damen E.J. (1988). Distribution of slow brain potentials related to motor preparation and stimulus anticipation in a time estimation task. Electroencephalogr. Clin. Neurophysiol..

[24] Böcker K.B.E., Baas J.M.P., Kenemans J.L., Verbaten M.N. (2001). Stimulus-preceding negativity induced by fear: A manifestation of affective anticipation. Int. J. Psychophysiol..

[25] Fuentemilla L., Cucurell D., Marco-Pallarés J., Guitart-Masip M., Morís J., Rodríguez-Fornells A. (2013). Electrophysiological correlates of anticipating improbable but desired events. NeuroImage.

[26] Craig A.D.B. (2009). How do you feel--now? The anterior insula and human awareness. Nat. Rev. Neurosci..

[27] Böcker K.B., Brunia C.H., van den Berg-Lenssen M.M. (1994). A spatiotemporal dipole model of the stimulus preceding negativity (SPN) prior to feedback stimuli. Brain Topogr..

[28] Brunia C.H.M., Hackley S.A., van Boxtel G.J.M., Kotani Y., Ohgami Y. (2011). Waiting to perceive: Reward or punishment?. Clin. Neurophysiol. Off. J. Int. Fed. Clin. Neurophysiol..

[29] Brunia C.H., de Jong B.M., van den Berg-Lenssen M.M., Paans A.M. (2000). Visual feedback about time estimation is related to a right hemisphere activation measured by PET. Exp. Brain Res..

[30] Sutton S., Braren M., Zubin J., John E.R. (1965). Evoked-potential correlates of stimulus uncertainty. Science.

[31] Nieuwenhuis S., Aston-Jones G., Cohen J.D. (2005). Decision making, the P3, and the locus coeruleus-norepinephrine system. Psychol. Bull..

[32] Vuillier L., Whitebread D., Szucs D. (2015). ERP evidence of cognitive strategy change in motivational conditions with varying level of difficulty. Neuropsychologia.

[33] Hughes G., Mathan S., Yeung N. (2013). EEG indices of reward motivation and target detectability in a rapid visual detection task. NeuroImage.

[34] Goldstein R.Z., Cottone L.A., Jia Z., Maloney T., Volkow N.D., Squires N.K. (2006). The effect of graded monetary reward on cognitive event-related potentials and behavior in young healthy adults. Int. J. Psychophysiol. Off. J. Int. Organ. Psychophysiol..

[35] Kleih S.C., Nijboer F., Halder S., Kübler A. (2010). Motivation modulates the P300 amplitude during brain-computer interface use. Clin. Neurophysiol. Off. J. Int. Fed. Clin. Neurophysiol..

[36] Yeung N., Sanfey A.G. (2004). Independent coding of reward magnitude and valence in the human brain. J. Neurosci. Off. J. Soc. Neurosci..

[37] Pessiglione M., Schmidt L., Draganski B., Kalisch R., Lau H., Dolan R.J., Frith C.D. (2007). How the brain translates money into force: A neuroimaging study of subliminal motivation. Science.

[38] Docx L., de la Asuncion J., Sabbe B., Hoste L., Baeten R., Warnaerts N., Morrens M. (2015). Effort discounting and its association with negative symptoms in schizophrenia. Cognit. Neuropsychiatry.

[39] Treadway M.T., Buckholtz J.W., Schwartzman A.N., Lambert W.E., Zald D.H. (2009). Worth the ‘EEfRT’? The Effort Expenditure for Rewards Task as an Objective Measure of Motivation and Anhedonia. PLoS ONE.

[40] Ubl B., Kuehner C., Kirsch P., Ruttorf M., Flor H., Diener C. (2015). Neural reward processing in individuals remitted from major depression. Psychol. Med..

[41] Chumbley J., Fehr E. (2014). Does general motivation energize financial reward-seeking behavior? Evidence from an effort task. PLoS ONE.

[42] Wolf D.H., Satterthwaite T.D., Kantrowitz J.J., Katchmar N., Vandekar L., Elliott M.A., Ruparel K. (2014). Amotivation in schizophrenia: Integrated assessment with behavioral, clinical, and imaging measures. Schizophr. Bull..

[43] St Onge J.R., Floresco S.B. (2009). Dopaminergic modulation of risk-based decision making. Neuropsychopharmacol. Off. Publ. Am. Coll. Neuropsychopharmacol..

[44] Floresco S.B., Tse M.T.L., Ghods-Sharifi S. (2008). Dopaminergic and glutamatergic regulation of effort- and delay-based decision making. Neuropsychopharmacol. Off. Publ. Am. Coll. Neuropsychopharmacol..

[45] Treadway M.T., Zald D.H. (2013). Parsing Anhedonia: Translational Models of Reward-Processing Deficits in Psychopathology. Curr. Dir. Psychol. Sci..

[46] Whitton A.E., Treadway M.T., Pizzagalli D.A. (2015). Reward processing dysfunction in major depression, bipolar disorder and schizophrenia. Curr. Opin. Psychiatry.

[47] Treadway M.T., Peterman J.S., Zald D.H., Park S. (2015). Impaired effort allocation in patients with schizophrenia. Schizophr. Res..

[48] Mata F., Treadway M., Kwok A., Truby H., Yücel M., Stout J.C., Verdejo-Garcia A. (2017). Reduced Willingness to Expend Effort for Reward in Obesity: Link to Adherence to a 3-Month Weight Loss Intervention. Obes. Silver Spring Md.

[49] Lawn W., Freeman T.P., Pope R.A., Joye A., Harvey L., Hindocha C., Mokrysz C., Moss A., Wall M.B., Bloomfield M.A. (2016). Acute and chronic effects of cannabinoids on effort-related decision-making and reward learning: An evaluation of the cannabis “amotivational” hypotheses. Psychopharmacology (Berl.).

[50] Gehring W.J., Willoughby A.R. (2002). The medial frontal cortex and the rapid processing of monetary gains and losses. Science.

[51] Holroyd C.B., Hajcak G., Larsen J.T. (2006). The good, the bad and the neutral: Electrophysiological responses to feedback stimuli. Brain Res..

[52] Plaisant O., Courtois R., Réveillère C., Mendelsohn G.A., John O.P. (2010). Validation par analyse factorielle du Big Five Inventory français (BFI-Fr). Analyse convergente avec le NEO-PI-R. Ann. Méd.-Psychol. Rev. Psychiatr..

[53] Fervaha G., Graff-Guerrero A., Zakzanis K.K., Foussias G., Agid O., Remington G. (2013). Incentive motivation deficits in schizophrenia reflect effort computation impairments during cost-benefit decision-making. J. Psychiatr. Res..

[54] Downloads - Cartool Community.

[55] Walentowska W., Paul K., Severo M.C., Moors A., Pourtois G. (2018). Relevance and uncertainty jointly influence reward anticipation at the level of the SPN ERP component. Int. J. Psychophysiol. Off. J. Int. Organ. Psychophysiol..

[56] Perrin F., Bertrand O., Pernier J. (1987). Scalp current density mapping: Value and estimation from potential data. IEEE Trans. Biomed. Eng..

[57] Cui J., Chen Y., Wang Y., Shum D.H.K., Chan R.C.K. (2013). Neural correlates of uncertain decision making: ERP evidence from the Iowa Gambling Task. Front. Hum. Neurosci..

[58] Bianchin M., Angrilli A. (2011). Decision Preceding Negativity in the Iowa Gambling Task: An ERP study. Brain Cogn..

[59] Hajcak G., Moser J.S., Holroyd C.B., Simons R.F. (2006). The feedback-related negativity reflects the binary evaluation of good versus bad outcomes. Biol. Psychol..

[60] Hajcak G., Holroyd C.B., Moser J.S., Simons R.F. (2005). Brain potentials associated with expected and unexpected good and bad outcomes. Psychophysiology.

[61] Bland A.R., Schaefer A. (2011). Electrophysiological correlates of decision making under varying levels of uncertainty. Brain Res..

[62] Chapman L.J., Chapman J.P., Raulin M.L. (1976). Scales for physical and social anhedonia. J. Abnorm. Psychol..

[63] Loas G., Dubal S., Perot P., Tirel F., Nowaczkowski P., Pierson A. (1997). [Validation of the French version of the Snaith-Hamilton Pleasure Scale (SHAPS, Snaith et al. 1995). Determination of the statistical parameters in 208 normal subjects and 103 hospitalized patients presenting with depression or schizophrenia]. L’Encéphale.

[64] Wardle M.C., Treadway M.T., Mayo L.M., Zald D.H., de Wit H. (2011). Amping up effort: Effects of d-amphetamine on human effort-based decision-making. J. Neurosci. Off. J. Soc. Neurosci..

[65] Kotani Y., Hiraku S., Suda K., Aihara Y. (2001). Effect of positive and negative emotion on stimulus-preceding negativity prior to feedback stimuli. Psychophysiology.

[66] Hillman C.H., Apparies R.J., Hatfield B.D. (2000). Motor and nonmotor event-related potentials during a complex processing task. Psychophysiology.

[67] Arulpragasam A.R., Cooper J.A., Nuutinen M.R., Treadway M.T. (2018). Corticoinsular circuits encode subjective value expectation and violation for effortful goal-directed behavior. Proc. Natl. Acad. Sci. USA.

[68] Wager T.D., Barrett L.F. (2017). From affect to control: Functional specialization of the insula in motivation and regulation. BioRxiv.

[69] Kennis M., Rademaker A.R., Geuze E. (2013). Neural correlates of personality: An integrative review. Neurosci. Biobehav. Rev..

[70] Deng Y., Li S., Zhou R., Walter M. (2018). Motivation but not valence modulates neuroticism-dependent cingulate cortex and insula activity. Hum. Brain Mapp..

[71] Ferdinand N.K., Kray J. (2013). Age-related changes in processing positive and negative feedback: Is there a positivity effect for older adults?. Biol. Psychol..

[72] Rigoni D., Polezzi D., Rumiati R., Guarino R., Sartori G. (2010). When people matter more than money: An ERPs study. Brain Res. Bull..

[73] Giustiniani J., Gabriel D., Nicolier M., Monnin J., Haffen E. (2015). Neural Correlates of Successful and Unsuccessful Strategical Mechanisms Involved in Uncertain Decision-Making. PLoS ONE.

[74] Polich J. (2007). Updating P300: An integrative theory of P3a and P3b. Clin. Neurophysiol. Off. J. Int. Fed. Clin. Neurophysiol..

[75] Polich J. (2004). Clinical application of the P300 event-related brain potential. Phys. Med. Rehabil. Clin. N. Am..

[76] Chase H.W., Swainson R., Durham L., Benham L., Cools R. (2010). Feedback-related Negativity Codes Prediction Error but Not Behavioral Adjustment during Probabilistic Reversal Learning. J. Cogn. Neurosci..

[77] Locke H.S., Braver T.S. (2008). Motivational influences on cognitive control: Behavior, brain activation, and individual differences. Cogn. Affect. Behav. Neurosci..

[78] Polezzi D., Sartori G., Rumiati R., Vidotto G., Daum I. (2010). Brain correlates of risky decision-making. NeuroImage.

[79] Takayoshi H., Onoda K., Yamaguchi S. (2018). Do Event-Related Evoked Potentials Reflect Apathy Tendency and Motivation?. Front. Hum. Neurosci..

[80] Dubal S., Pierson A., Jouvent R. (2000). Focused attention in anhedonia: A P3 study. Psychophysiology.

[81] Horovitz S.G., Skudlarski P., Gore J.C. (2002). Correlations and dissociations between BOLD signal and P300 amplitude in an auditory oddball task: A parametric approach to combining fMRI and ERP. Magn. Reson. Imaging.

[82] Wang Y., Deng Y., Sui D., Tang Y.-Y. (2014). Neural correlates of cultural differences in moral decision making: A combined ERP and sLORETA study. Neuroreport.

[83] Yacubian J., Gläscher J., Schroeder K., Sommer T., Braus D.F., Büchel C. (2006). Dissociable systems for gain- and loss-related value predictions and errors of prediction in the human brain. J. Neurosci. Off. J. Soc. Neurosci..

[84] Rogers R.D., Ramnani N., Mackay C., Wilson J.L., Jezzard P., Carter C.S., Smith S.M. (2004). Distinct portions of anterior cingulate cortex and medial prefrontal cortex are activated by reward processing in separable phases of decision-making cognition. Biol. Psychiatry.

[85] Raymond J. (2009). Interactions of attention, emotion and motivation. Prog. Brain Res..

[86] Umemoto A., Holroyd C.B. (2017). Neural mechanisms of reward processing associated with depression-related personality traits. Clin. Neurophysiol. Off. J. Int. Fed. Clin. Neurophysiol..

[87] Salamone J.D., Correa M., Farrar A., Mingote S.M. (2007). Effort-related functions of nucleus accumbens dopamine and associated forebrain circuits. Psychopharmacology (Berl.).

[88] Wade T.R., de Wit H., Richards J.B. (2000). Effects of dopaminergic drugs on delayed reward as a measure of impulsive behavior in rats. Psychopharmacology (Berl.).

[89] Massar S.A.A., Libedinsky C., Weiyan C., Huettel S.A., Chee M.W.L. (2015). Separate and overlapping brain areas encode subjective value during delay and effort discounting. NeuroImage.

[90] Winstanley C.A., Theobald D.E.H., Dalley J.W., Cardinal R.N., Robbins T.W. (2006). Double dissociation between serotonergic and dopaminergic modulation of medial prefrontal and orbitofrontal cortex during a test of impulsive choice. Cereb. Cortex N.Y. N 1991.

[91] Schuermann B., Endrass T., Kathmann N. (2012). Neural correlates of feedback processing in decision-making under risk. Front. Hum. Neurosci..

[92] Gheza D., De Raedt R., Baeken C., Pourtois G. (2018). Integration of reward with cost anticipation during performance monitoring revealed by ERPs and EEG spectral perturbations. NeuroImage.

[93] Protopopescu X., Pan H., Altemus M., Tuescher O., Polanecsky M., McEwen B., Silbersweig D., Stern E. (2005). Orbitofrontal cortex activity related to emotional processing changes across the menstrual cycle. Proc. Natl. Acad. Sci. USA.

[94] Tanner D., Morgan-Short K., Luck S.J. (2015). How inappropriate high-pass filters can produce artifactual effects and incorrect conclusions in ERP studies of language and cognition. Psychophysiology.

